# Influence of the screw-home mechanism on TT-TG distance: a 3D kinematic simulation study

**DOI:** 10.1007/s00590-026-04708-y

**Published:** 2026-03-09

**Authors:** Maximilian Jörgens, Julian Fürmetz, Markus Bormann, Wolfgang Böcker, Boris Michael Holzapfel, Sebastian Siebenlist, Julius Watrinet

**Affiliations:** 1 Department of Orthopaedics and Trauma Surgery, Musculoskeletal University Center Munich (MUM), University Hospital, LMU, Munich, Germany; 2 Department of Trauma Surgery, BG Trauma Center Murnau, Murnau am Staffelsee, Germany; 3 Department of Orthopaedic Sports Medicine, Technical University of Munich, Munich, Germany; 4 endogap - Joint Replacement Institute, Garmisch-Partenkirchen Medical Center, Garmisch-Partenkirchen, Germany

**Keywords:** TT-TG, Screw-home mechanism, Knee kinematics, 3D simulation

## Abstract

**Background:**

The axial distance between the tibial tuberosity and the trochlear groove (TT-TG) is a significant parameter in the preoperative analysis of patellofemoral instability. Discrepancies in TT-TG measurements between imaging modalities are largely attributed to differences in knee flexion and the associated tibial rotation (screw-home mechanism). However, the quantitative impact of the screw-home mechanism and its inter-individual variability on TT-TG measurement remains incompletely characterized. The aim of this study was to measure and evaluate the influence of the screw-home mechanism during knee flexion on the TT-TG distance.

**Methods:**

56 computed tomography (CT) scans were obtained and segmented into surface models. Anatomic landmarks were defined to determine the TT-TG. Kinematic simulations of knee flexion (0–30° in 10° increments) were performed. Concurrently, various combined screw-home mechanisms were performed in 5° increments (0–20°) for each model. Data analysis involved the paired samples Friedman test with post-hoc Wilcoxon signed-rank tests (*p* < 0.05).

**Results:**

Flexion and internal tibial rotation led to significant changes in TT-TG values (*p* < 0.001). Post-hoc analyses showed that all pairwise comparisons within a degree of flexion were statistically significant at different rotation angles. Knee flexion resulted in minimal changes in TT-TG distance, whereas the additional screw-home mechanism caused significant deviations depending on the degree of flexion and rotation, ranging from -1.5 mm (± 0.3) to − 11.0 mm (± 1.1). Once the rotation was complete, further flexion increased the TT-TG distance.

**Conclusion:**

The TT-TG distance is significantly influenced by the screw-home mechanism. Standardizing the TT-TG assessment at 30° of flexion minimizes rotational variability, potentially improving diagnostic consistency. However, in vivo validation is required before clinical implementation.

## Introduction

Patellofemoral instability (PFI) relies on a complex interplay between osseous morphology and soft-tissue restraints [1, 2]. Several anatomical risk factors for patellofemoral instability have been identified, including trochlear dysplasia, patella alta or malalignment of the tibial tuberosity [1]. Among these variables, the tibial tuberosity-trochlear groove (TT-TG) distance has emerged as a key radiographic parameter, as it quantifies the lateralizing vector acting on the patella and thus stratifies the risk for first‑time or recurrent dislocations. Indeed, patients with PFI consistently display higher TT-TG values compared to controls [3, 4]. In the context of preoperative planning of medializing tibial tuberosity osteotomy, TT-TG measurement provides guidance for patient selection and correction magnitude [2]. The recommended cutoff values differ depending on the imaging modality, with approximately 20 mm for computed tomography (CT) measurements and 13 mm for magnetic resonance imaging (MRI) [2].

CT and MRI are both commonly used to measure TT-TG, with MRI typically performed with the knee flexed at approximately 30° and CT usually acquired in full extension (0° flexion) [5–9]. Although CT has been reported to yield higher TT-TG values, this is not inherent to the modality. Discrepancies largely reflect differences in knee positioning rather than the imaging technique. Consequently, distinct cutoff values for TT-TG have been proposed for each modality, and it should be noted that MRI can also be performed with the knee extended, which must be considered when interpreting cutoff values [9, 10].

During knee extension, there is also tibial external rotation, and together, this is described as the screw-home mechanism. This mechanism involves tibial external rotation relative to the femur during the final 10–15° of knee extension, enhancing knee joint stability [11]. Differences in TT-TG distances observed between imaging modalities have been attributed, in part, to this rotational motion [12, 13]. The screw-home mechanism shows considerable inter-individual variability, as both the magnitude and timing of tibial external rotation depend on whether the movement is active, passive, loaded or unloaded [11]. Notably, the screw-home mechanism increases abruptly during the final degrees of active extension, whereas it appears more gradual and reduced during passive or non-weight-bearing motion, and is markedly diminished in pathological conditions such as knee osteoarthritis [11, 14]. As previously described by Müller, the asymmetric geometry of the femoral condyles is the primary causal factor and plays a decisive role in driving the screw-home mechanism [15].

These variations underscore the need for a more comprehensive investigation of the screw-home mechanism to clarify its biomechanical and clinical relevance, particularly since its incomplete characterization may influence radiologic parameters such as the TT-TG distance.

To address this gap, the present study uses CT-based 3D (three-dimensional) models and computer-assisted simulations to systematically investigate the relationships among tibiofemoral flexion, screw-home mechanism and the TT-TG distance. It was hypothesized that an increased tibial internal rotation would correlate negatively with lower TT-TG values.

## Material & methods

### Study population

**Fig. 1:**
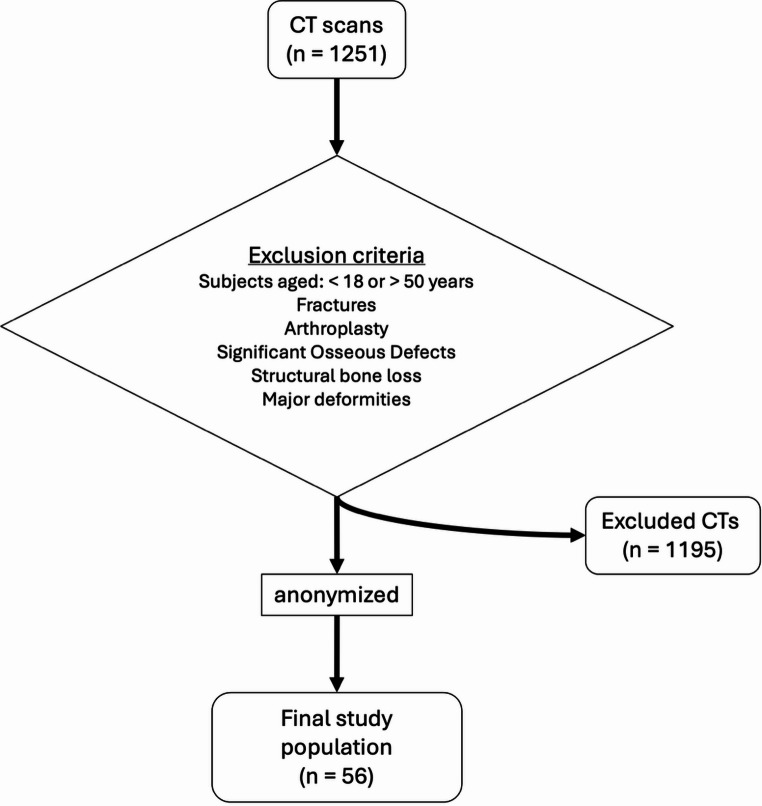
Flowchart of the study population selection process. From an initial retrospective dataset of 1251 post-mortem whole-body CT scans, 56 scans were selected for simulation

The institutional review board approved this study (EC-Nr.: 17-044), which was designed as a simulation study. The reporting of this study follows the STROBE (Strengthening the Reporting of Observational Studies in Epidemiology) guidelines. Post-mortem whole-body CT scans were retrospectively analyzed. From an initial dataset of 1251 available scans, 56 lower-limb CT scans were selected based on exclusion criteria (Fig. 1). Exclusion criteria included fractures, arthroplasty, significant osseous defects (precluding landmark identification), and age < 18 or > 50 years (to ensure skeletal maturity and minimize degenerative changes).

### Data preparation

CT scans (GE HD750, GE Healthcare) were performed in a supine position using helical mode (120 kV, 100–650 mA, Noise Index 8.84, 0.8 s rotation time, 0.984:1 pitch). Images were reconstructed with a maximum slice thickness of 1.25 mm using a bone kernel. Adherence to these parameters and spatial accuracy were ensured through the scanner’s internal calibration and routine quality assurance, meeting manufacturer specifications for high-resolution imaging. The resulting data were processed to reconstruct accurate 3D bone models using Slicer software (version 3.5.0; https://www.slicer.org) [16]. An experienced orthopedic surgeon established a standardized global coordinate system and anatomical landmarks for measuring the TT-TG using Blender software (version 3.5.1; Blender Foundation, Amsterdam, Netherlands), as previously validated [17, 18]. The anatomical landmarks employed demonstrated high intra- and interobserver reliability [17, 18]. The coordinate system was established by defining the X-axis through the best-fit cylinder encompassing both femoral condyles. The Z-axis was then determined by drawing a line orthogonal to the X-axis, intersecting through the femoral head center (FHC), consistent with previously published methodology [19, 20]. The Y-axis was defined perpendicular to both the X- and Z-axes, completing the orthogonal coordinate system. This approach resulted in precise 3D models of femoral and tibial anatomy, which closely aligned with established simulation techniques and previously reported mathematical validations [21].

### Screw-home mechanism

The screw-home mechanism involves external tibial rotation during terminal extension (10–15°), driven primarily by the asymmetric geometry of the femoral condyles [15, 22]. Clinically, this rotation is critical for TT-TG assessment, as it directly alters the spatial relationship between the tibial tuberosity and the trochlea.

**Fig. 2:**
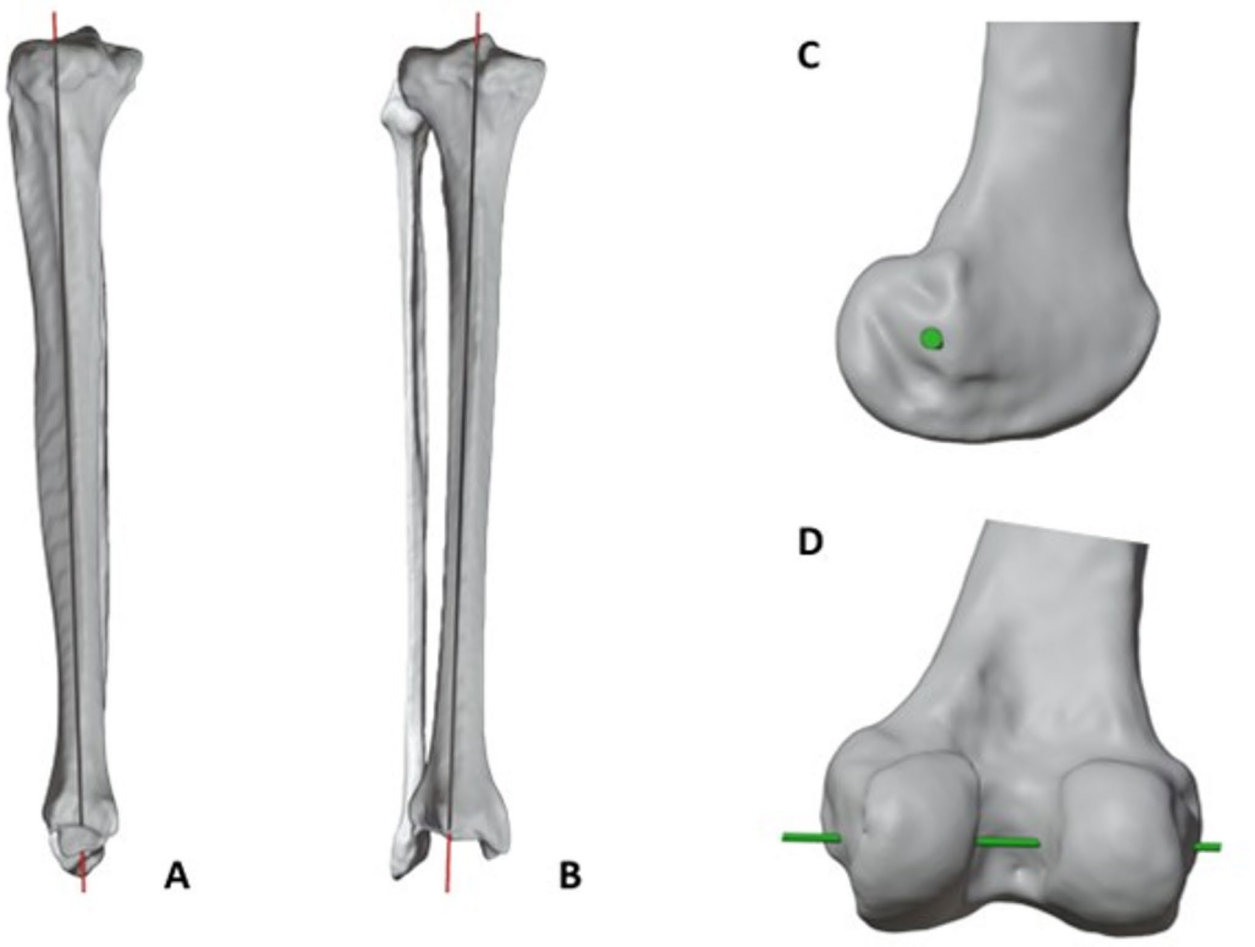
Illustration of the axes of rotation as the cause of the screw-home mechanism; longitudinal rotation of the tibia (red axis = tibial axial rotation (TAR); lateral view (**A**), coronal view (**B**)), flexion of the knee (green axis = femoral axial rotation (FAR); lateral view (**C**), posterior view (**D**))

To simulate this influence, two axes were defined: The femoral axial rotation (FAR), representing the flexion–extension axis, was centered through a best-fit cylinder of the femoral condyles to approximate the surgical transepicondylar axis. The tibial axial rotation (TAR), used to simulate internal rotation, was derived from the anatomical tibial axis by connecting validated proximal (TAPP: Midpoint 1/3 down the tibial shaft) and distal tibial shaft midpoints (TADP; Midpoint 2/3 down the tibial shaft) (Fig. 2) [18]. To minimize observer bias and measurement error, all landmark placements and measurements were performed and verified by an orthopedic surgeon following a standardized protocol.

Although CT acquisition occurred in full extension, this position does not guarantee maximal tibial external rotation, as rotational alignment in the supine position is influenced by soft-tissue resting tension. Nevertheless, for the purpose of analysis, it was assumed that the tibia exhibited some degree of external rotation in this extended position. Knee flexion was simulated via incremental rotation of the tibia around the FAR axis (0° to 30° in 10° steps), while concomitant internal tibial rotation was applied around the TAR axis (0° to 20° in 5° steps). The range of the screw-home mechanism and knee flexion were chosen to capture tibial rotation, reflecting external rotation in full extension and progressive internal rotation during early flexion and to capture differences between image techniques (CT vs. MRI) [5, 11]. This dual-axis approach generated five simulation groups reflecting varying degrees of tibiofemoral kinematics. A Python script was used for simulation, resulting in a comprehensive dataset (Version 3.11, Python Software Foundation, Wilmington, DE, USA).

### TT-TG measurement

**Fig. 3:**
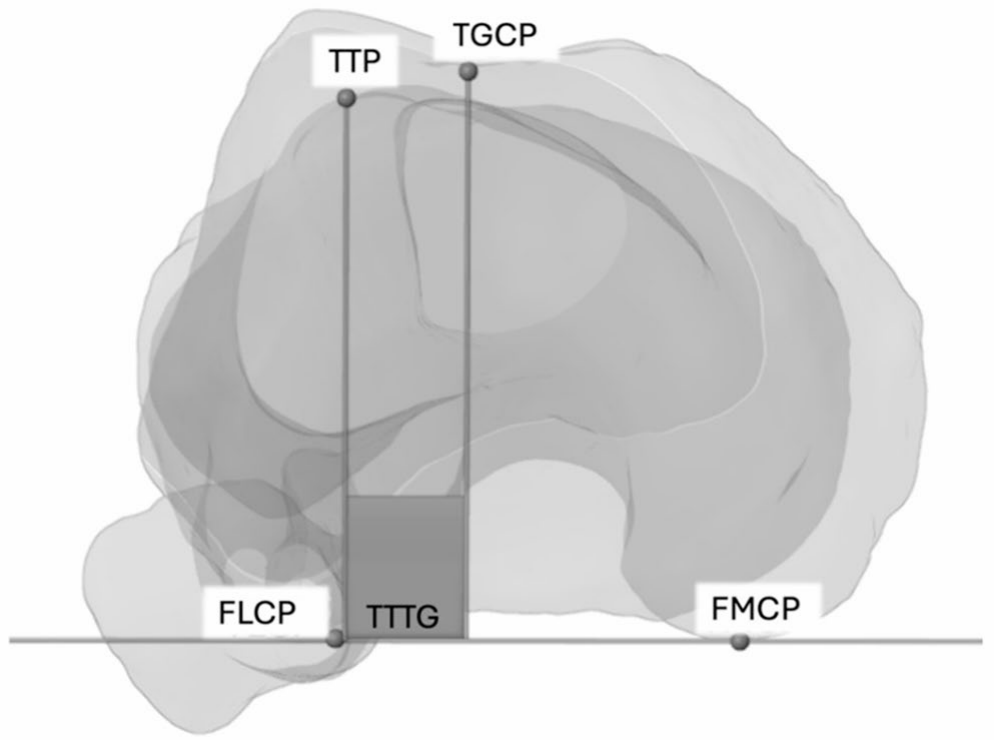
Determination of the TT-TG through FMCP and FLCP, the TTP and the TGCP; FMCP: medial posterior femoral condyle, FLCP: lateral posterior femoral condyle, TTP: tibial tuberosity, TGCP: trochlear fossa

The TT-TG distance was measured on axial CT images using the established technique based on the anatomical landmarks of the tibial tuberosity (TTP), the trochlear groove centre point (TGCP), and the medial and lateral posterior femoral condyles (FMCP, FLCP) [23]. The FMCP, FLCP, and TGCP defined the XY-plane of the global femoral coordinate system. The TTP and TGCP were then orthogonally projected onto the posterior condylar axis (FMCP-FLCP), and the linear separation between these projected points was recorded as the axial TT-TG distance (Fig. 3).

### Statistical analysis

As the simulation protocols were performed on complete CT datasets, there were no missing data, and no imputation methods were required. First, data were tested for normal distribution using the Shapiro–Wilk test. Descriptive statistics, including means and standard deviations (SD), were calculated for all variables. A series of one-way repeated measures analyses, employing the appropriate paired samples Friedman test, were conducted to isolate the main effect of each factor. Specifically, the effect of rotation was tested at a fixed flexion angle. Pairwise post-hoc analyses were made using the Wilcoxon signed-rank test, with adjustments for multiple comparisons using the Bonferroni correction. Finally, the results were tested for statistical significance (*p* < 0.05). Data analysis was conducted using IBM SPSS Statistics (Version 29, IBM Corp., Armonk, NY, USA) and Python (Version 3.11, Python Software Foundation, Wilmington, DE, USA). Visualizations were generated using the Matplotlib library (Hunter JD, open source).

## Results

A total of 56 lower-limb CT scans were included in the final analysis. Due to the anonymized nature of the retrospective dataset, specific demographic details beyond the inclusion criteria were not available. However, all included subjects were aged between 18 and 50 years to ensure skeletal maturity and minimize the influence of advanced degenerative changes on the osseous landmarks.

Firstly, the alterations in TT-TG values were analyzed in flexion without rotation. At baseline (0° flexion, 0° rotation), the mean TT-TG distance was 14.1 mm (± 4.0 mm). Isolated knee flexion without screw-home mechanism resulted in negligible changes to TT-TG values; these variations were not statistically significant (*p* > 0.1; Table 1).

**Table 1:** Changes in TT-TG distance depending on the flexion of the knee (no tibial rotation); SD: standard deviation, TT-TG: tibial tuberosity-trochlear groove

Flexion (°)	Mean TT-TG (mm)	SD (mm)	*p*-value
0	14.1	4.0	–
10	14.1	4.0	0.144
20	14.1	4.0	0.233
30	14.0	4.0	0.311

The influence of screw-home mechanism during flexion on the TT-TG values was analyzed within the range of 30° flexion. With a flexion of 10° and an internal rotation of 5°, the TT-TG value decreased to 11.5 ± 4.0 mm compared to the initial condition. This reduction was statistically significant (*p* < 0.001), with the magnitude of the effect increasing proportionally with the degree of internal rotation (Table 2).

**Table 2:** Mean values of the TT-TG distance (mm) with standard deviation (SD) and 95% confidence interval (CI); TT-TG: tibial tuberosity-trochlear groove

Internal tibial rotation (°)	0	5	10	15	20
0° knee flexion	14.1 (± 4.0) [13.0, 15.1]	–	–	–	–
10° knee flexion	14.1 (± 4.0) [13.0, 15.1]	11.5 (± 4.0) [10.4, 12.5]	8.8 (± 4.0) [7.7, 9.8]	5.9 (± 4.0) [4.9, 7.0]	3.0 (± 4.0) [2.0, 4.1]
20° knee flexion	14.1 (± 4.0) [13.0, 15.1]	12.0 (± 4.0) [10.9, 13.1]	9.8 (± 4.0) [8.7, 10.9]	7.5 (± 4.0) [6.4, 8.6]	5.1 (± 4.0) [4.0, 6.2]
30° knee flexion	14.0 (± 4.0) [13.0, 15.1]	12.6 (± 4.0) [11.5, 13.6]	10.9 (± 4.0) [9.9, 12.0]	9.2 (± 4.0) [8.1, 10.3]	7.3 (± 4.0) [6.3, 8.4]

**Fig. 4:**
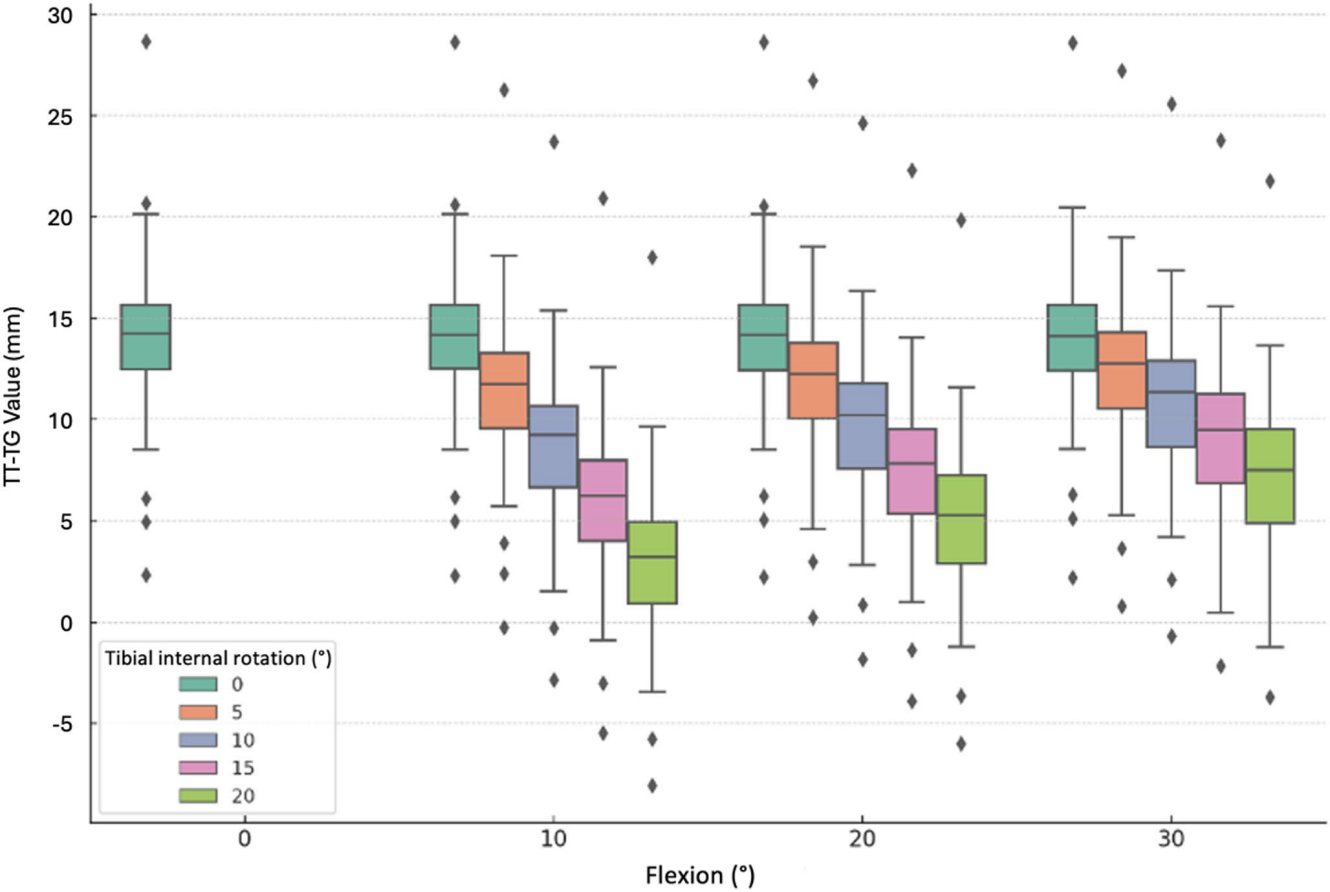
TT-TG values and changes as affected by knee internal rotation and grouped by knee flexion; Presentation of distribution across 10°, 20° and 30° of knee flexion. For each internal tibial rotation a color-coding boxplot. Representation of baseline TT-TG values (zero rotation) by the teal boxplots: TT-TG: tibial tuberosity-trochlear groove

Figure 4 shows different degrees of screw-home mechanism with increasing flexion, grouped according to different rotations (X-axis). The teal box plot represents the initial value of TT-TG with the leg unrotated and extended.

TT-TG changes (ΔTT-TG) caused by screw-home mechanism, compared to the baseline condition (unrotated tibia), were all significant in pairwise comparisons (Wilcoxon test with Bonferroni correction, *p* < 0.001). Table 3 presents ΔTT-TG values for variations in the screw-home mechanism across varying degrees of flexion. Notably, even small amounts of flexion and rotation (e.g., 10° flexion and 5° rotation) lead to significant changes in TT-TG. Relative analyses (not further shown) indicate that this combination already produces a mean change of 21.2% (± 13.9; 95% CI − 24.9; − 17.4), highlighting the high sensitivity of TT-TG to minimal rotational deviations and explaining the significant differences observed even for small absolute displacements.

**Table 3:** Changes in TT-TG (ΔTT-TG) compared to the unrotated tibia with standard deviation (SD) and 95% confidence interval (CI); TT-TG: tibial tuberosity-trochlear groove

Internal tibial rotation (°)	5	10	15	20
10° knee flexion	− 2.6 (± 0.3) [− 2.7, − 2.5]	− 5.3 (± 0.5) [− 5.5, − 5.2]	− 8.1 (± 0.8) [− 8.3, − 7.9]	− 11.0 (± 1.1) [− 11.3, − 10.8]
20° knee flexion	− 2.1 (± 0.3) [− 2.1, − 2.0]	− 4.2 (± 0.5) [− 4.4, − 4.1]	− 6.6 (± 0.8) [− 6.8, − 6.3]	− 9.0 (± 1.0) [− 9.2, − 8.7]
30° knee flexion	− 1.5 (± 0.3) [− 1.6, − 1.4]	− 3.1 (± 0.5) [− 3.3, − 3.0]	− 4.9 (± 0.8) [− 5.1, − 4.6]	− 6.7 (± 1.1) [− 7.0, − 6.4]

As shown in Table 3, ΔTT-TG progressively decreased with increasing knee flexion in the absence of additional internal tibial rotation after the first 10° of flexion. Variability in ΔTT-TG was markedly reduced at 30° of knee flexion relative to the 10° position.

## Discussion

The principal finding of this study is that the screw-home mechanism significantly alters TT-TG distance, demonstrating a strong negative correlation that is modulated by the degree of knee flexion. Furthermore, internal rotation demonstrated a strong negative correlation with TT-TG, whereas flexion showed a weaker, yet still positive, correlation.

The TT-TG distance is a widely used parameter for the classification of patellar instability [24, 25]. The lateralized tibial tuberosity is one of the risk factors of patellar instability [5, 26–28]. Radiological imaging, whether CT or MRI, plays a central role in clinical decision-making. While CT has primarily been used to determine TT-TG, MRI interpretation of TT-TG has been increasingly studied and applied in clinical practice in recent years [5, 23]. The TT-TG limit of 15 or 20 mm in the extended position is still widely used, but there is still significant variability in defining physiological cutoffs [10, 23, 29].

The observed reduction in TT-TG distance during knee flexion and tibial internal rotation can be attributed to the complexity of the knee joint’s biomechanics, including mechanisms such as the screw-home mechanism [30]. During flexion, relative movement occurs between the femur and the tibia, resulting in a change in the alignment of the tibial tuberosity relative to the trochlear groove [31, 32]. The reduced variability of the TT-TG distance observed at 30° of knee flexion may additionally be explained by the closer approximation between the posterior femoral condyles and the tibial plateau at this angle, which stabilizes tibiofemoral alignment and thereby limits rotational divergence. This mechanism has been found to be challenging to quantify precisely due to its dependence on factors such as the load on the lower leg and the nature of the movement, whether active or passive [11, 33, 34]. In the present simulation, a pronounced change in TT-TG was observed in response to tibial internal rotation. The lower limb alignment during supine CT imaging may not correspond to a fully externally rotated tibial position, as rotation can vary with the subject’s relaxation. If the initial tibial position is less than full external rotation, applying internal rotation in the simulation could artificially reduce TT-TG values. This uncertainty in the starting position introduces a potential source of bias that should be considered when interpreting the magnitude of simulated TT-TG changes. Although conclusions regarding the absolute magnitude can be drawn, the findings indicate that with increasing knee flexion the trend of TT-TG variation attenuates and tends to shift toward higher TT-TG values.

The simulated values obtained in this study—7.3 mm ± 4.0 (95% CI 6.3–8.4) at 30° flexion with 20° internal tibial rotation and 14.1 mm ± 4.0 (95% CI 13.0–15.1) at full extension—show strong agreement with the in vivo measurements reported by Dietrich et al. (8.1 mm ± 3.4 at 30° flexion and 15.1 mm ± 3.2 at full extension) [13]. This congruence with in vivo measurements supports the physiological plausibility and geometric validity of the simplified simulation model, suggesting that it can yield consistent TT-TG estimates even without incorporating dynamic loading or soft-tissue constraints. In the context of TT-TG changes, it is also important to consider the knee rotation angle [35]. This angle, defined by the posterior condylar axis of the femur and the tangent to the posterior tibial plateau, represents the relative axial rotation of the tibia in relation to the femur. It has been demonstrated that this rotational alignment influences TT-TG measurements, as well as potential osseous or cartilage-related changes [14, 30]. In their study, Ackermann et al. showed that the knee rotation angle contributes to the physiological screw-home mechanism and thereby affects the lateralization of the tibial tuberosity. However, they also demonstrated that this effect is less pronounced in pathological knee rotation angles (> 20° external rotation of the tibia relative to the femur), resulting in a smaller-than-expected influence on TT-TG compared with the native knee [30].

The results of this study show that the tibial internal rotation and knee flexion result in a decrease of the TT-TG distance, depending on their magnitude. This finding is consistent with a previous in vivo study that has also documented a decrease in TT-TG distance with increasing flexion [13]. Tan et al. found in their study that the TT-TG of patellofemoral stable patients was 12.85 mm (95% CI 11.71–14.01) on CT and 9.83 mm (95% CI 9.11–10.54) on MRI [9].

The present study corroborates and extends previous findings by quantitatively measuring the direct influence of rotation on TT-TG distance with greater precision. Clinical observations of TT-TG changes due to knee flexion and rotation were confirmed, while simulations improved understanding of these effects at different degrees of motion. Notably, significant TT-TG changes were observed even with mild flexion and rotation, highlighting the influence of screw-home mechanism during knee flexion.

These findings have important clinical implications. Systematic discrepancies in TT-TG measurements between CT and MRI have been repeatedly reported and are commonly attributed to differences in knee positioning during image acquisition, particularly with respect to knee flexion [36]. The present simulation demonstrates that these discrepancies are not solely positional but are strongly influenced by tibial rotation associated with the screw-home mechanism, which is most pronounced near full extension.

Because the magnitude and timing of this rotational component vary considerably between individuals and cannot be reliably assessed by clinical examination or standard static imaging, TT-TG measurements obtained close to full extension are inherently susceptible to rotational variability. Measuring the TT-TG distance at 30° of knee flexion may reduce this variability by positioning the knee beyond the phase of greatest rotational change, thereby improving measurement consistency and comparability across imaging modalities.

Standardizing the measurement at 30° of flexion offers a promising opportunity to enhance diagnostic consistency and develop flexion-specific reference values. The simulated data in this study support this potential, as the measured values align well with established literature [13]. Although the interpretation of absolute values is influenced by the assumed initial rotational state in static imaging, and the broad simulation range may exceed typical physiological conditions in some cases, this approach provides a robust exploratory framework. Further in vivo validation remains the next logical step to confirm these findings and establish their full clinical and surgical relevance.

### Limitations

A key limitation of the present study lies in its reliance on surface-based rigid-body simulations, which do not account for dynamic soft-tissue behavior. Consequently, ligamentous constraints, muscular forces, and joint contact mechanics—critical contributors to physiological knee motion—are not represented. Incorporating these factors would require patient-specific modeling of ligament properties, muscle activation patterns, and contact mechanics, which are currently infeasible due to limitations in variability between individuals. Furthermore, knee joint kinematics were simplified by assuming a fixed-axis rotation, whereas the true motion involves complex combinations of rolling, gliding, pivoting and rotation governed by anatomical and biomechanical factors. These simplifications inherently limit the ability to replicate in vivo conditions. Nonetheless, the primary aim of this study was to isolate and quantify the influence of knee flexion and internal rotation on TT-TG distance. In this context, the chosen simulation framework provides a controlled and reproducible means of evaluating these geometric changes, while acknowledging its limitations in reflecting the full physiological complexity of the joint. A further limitation is the considerable inter-individual variability in the extent and timing of the screw-home mechanism, which cannot be fully captured by the standardized simulation parameters used in this study. Given these limitations, supplementary in vivo studies should be considered.

## Conclusion

The TT-TG distance is significantly influenced by the screw-home mechanism. Standardizing the TT-TG assessment at 30° of flexion minimizes rotational variability, potentially improving diagnostic consistency. However, in vivo validation is required before clinical implementation.

## Data Availability

No datasets were generated or analysed during the current study.
